# A Novel Mutation in Helical Domain 2 of *NOD2* in Sporadic Blau Syndrome

**DOI:** 10.1080/09273948.2016.1207789

**Published:** 2016-09-13

**Authors:** Lubhani Jain, Namrata Gupta, Mamatha M. Reddy, Ruchi Mittal, Manas Ranjan Barik, Bharat Panigrahi, Tom Monie, Soumyava Basu

**Affiliations:** ^a^ Retina and Uveitis Service, LV Prasad Eye Institute, Bhubaneswar, India; ^b^ Biology Service, LV Prasad Eye Institute, Bhubaneswar, India; ^c^ Dalmia Ophthalmic Pathology Service, LV Prasad Eye Institute, Bhubaneswar, India; ^d^ Internal Medicine Service, L V Prasad Eye Institute, Bhubaneswar, India; ^e^ MRC Human Nutrition Research, Elsie Widdowson Laboratory, Cambridge, UK

## Abstract

We report a 12-year-old girl who presented with bilateral granulomatous anterior uveitis accompanied by boggy arthritis of knee and ankle joints, intermittent fever, and nodular skin rash. She was diagnosed with sporadic Blau syndrome (early-onset sarcoidosis) based on above clinical signs and presence of non-necrotising granuloma on iris biopsy. DNA sequencing revealed a previously unreported heterozygous mutation consisting of a G>A transition in exon 4 of the *NOD2* gene. This resulted in a glutamic acid to lysine substitution in helical domain 2 of the nucleotide binding and oligomerization (NACHT) region, possibly reducing efficiency of auto-inhibition in NOD2 signaling. Interestingly, the ocular inflammation resolved completely following therapeutic vitrectomy in both eyes whereas the systemic symptoms of fever and arthritis continued to wax and wane while on treatment with oral methotrexate and corticosteroids.

## INTRODUCTION

Blau syndrome (BS), first described by Blau and Jabs,^1-^ ^2^ is a rare, autosomal dominant, monogenic autoinflammtory syndrome caused by mutation(s) in the *NOD2* gene. Familial BS and its sporadic variant early-onset sarcoidosis (EOS) consist of a triad of dermatitis, arthritis, and uveitis. Ocular manifestations include anterior uveitis, multifocal choroiditis, and panuveitis appearing around 4 years of age.^3^ Treatment with corticosteroids and immunosuppressive is not always effective and long-term prognosis is unknown.^3^ We report a case of sporadic BS (EOS) with a novel *NOD2* mutation.

## CASE DESCRIPTION

**FIGURE 1.**
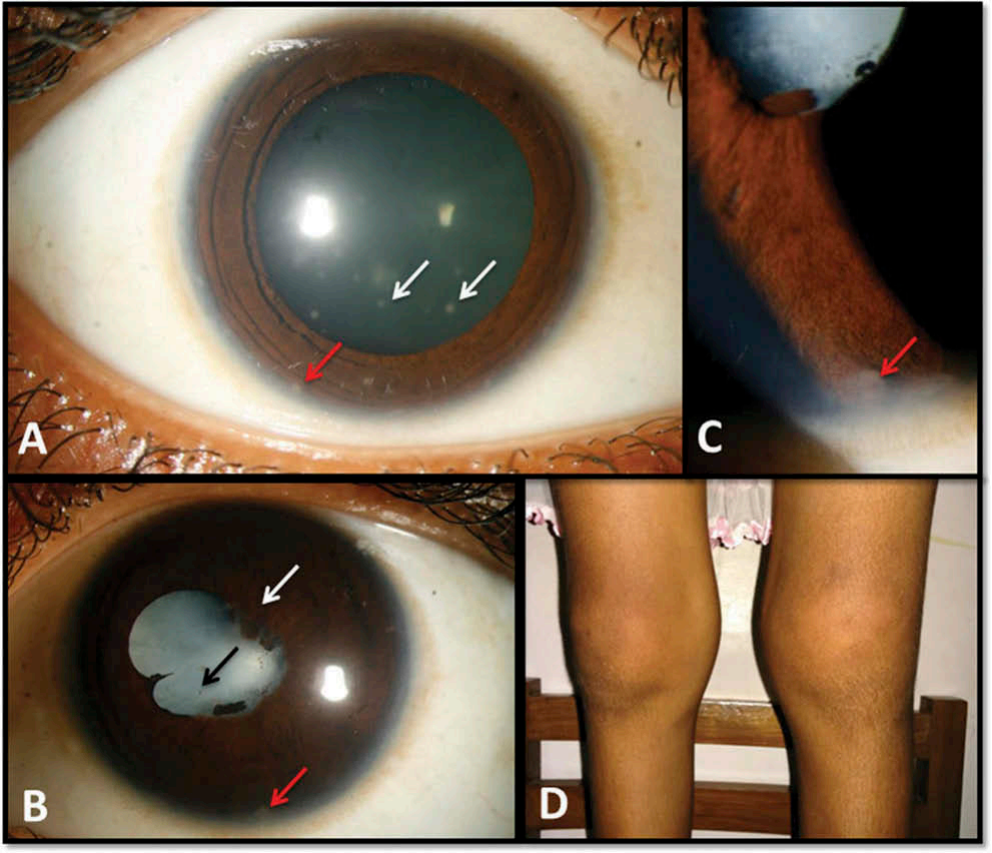
Clinical photographs. (A) Right eye: keratic precipitates (white arrows), iris nodules (red arrow). (B) Left eye: posterior synechiae (white arrow), complicated cataract (black arrow), iris nodule (red arrow). (C) Left eye: iris nodules (red arrow), magnified image. (D) Swelling of knee joints.

A 12-year-old girl presented with sudden, painful defective vision in the right eye since 1 month and decreased vision in the left eye since 3 years. Her best corrected visual acuity (BCVA) was counting fingers and hand movements in the right and left eye respectively. The right eye showed non-granulomatous anterior uveitis with vitreous haemorrhage (Figure 1A) and the left eye showed chronic anterior uveitis, multiple iris granuloma, and complicated cataract (Figure 1B-C). She suffered from intermittent febrile episodes and a nodular rash over the legs since 3 years of age and painful, boggy swelling of the knee and ankle joints for the past 6 months (Figure 1D). Chest imaging, tuberculin skin test, and anti-nuclear antibodies were negative, serum angiotensin converting enzyme levels were elevated, and ultrasonography of the abdomen revealed hepato-splenomegaly. There was no family history of similar disease. Based on the above history and clinical findings, we diagnosed sporadic BS (EOS).

**FIGURE 2.**
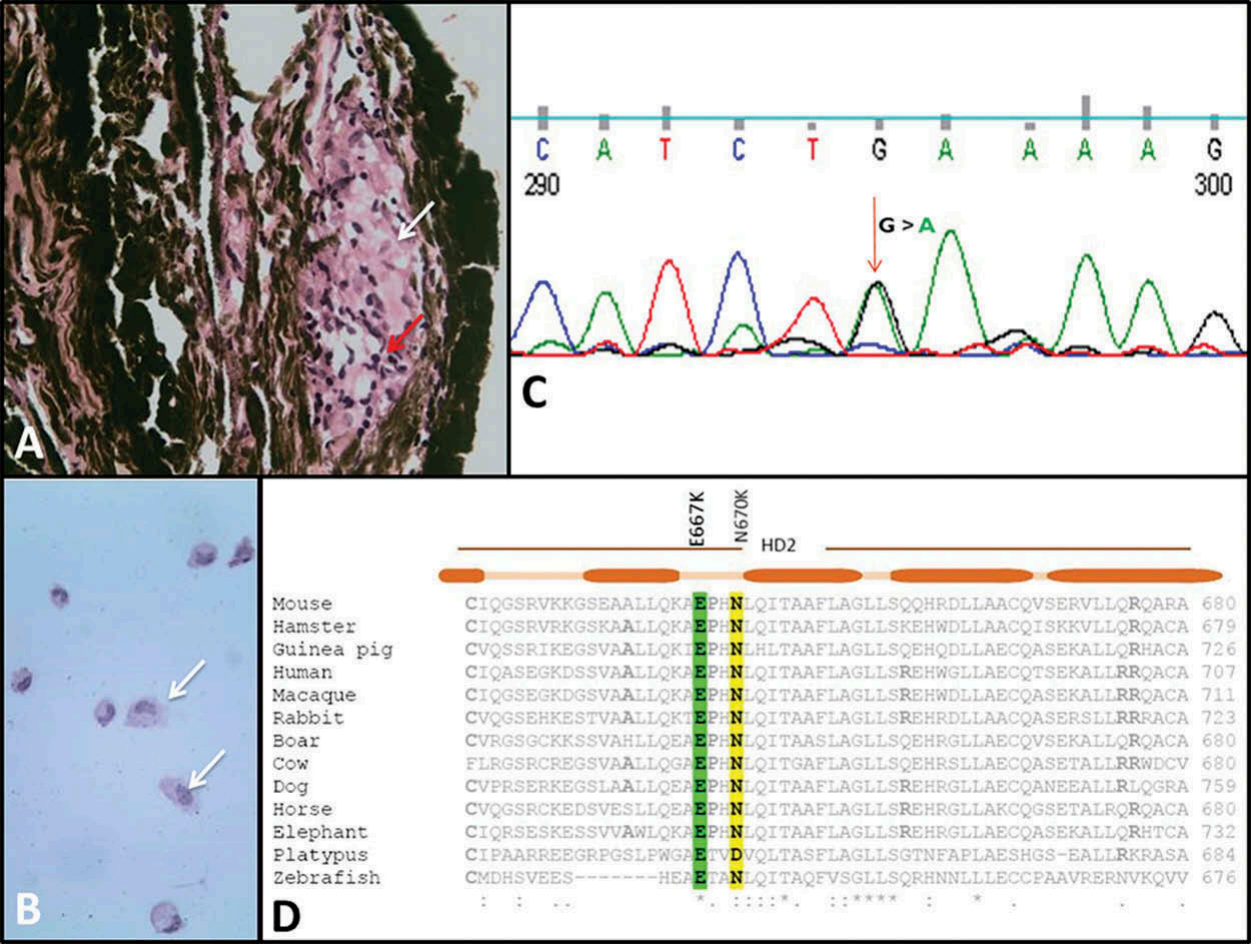
Histopathology and DNA sequencing. (A) Left eye iris nodule biopsy: non-caseating granuloma, histiocytes (white arrow), lymphocytes (red arrow). (B) Right eye vitreous: histiocytes (white arrows). (C) G>A transition in exon 4 of *NOD2*. (D) Multiple sequence alignment of E667 in helical domain 2 of NACHT in *NOD2*.

The patient was treated with oral steroids and methotrexate. She underwent pars plana vitrectomy with anterior chamber (AC) tap in the right eye and iris granuloma biopsy followed by cataract surgery and therapeutic vitrectomy in the left eye. At final follow-up after 8 months, her BCVA was 20/30 and 20/25 in the right and left eye respectively with no active ocular inflammation. Her nodular skin rash also resolved but the systemic inflammatory signs of fever and arthritis continued to wax and wane while on treatment with oral corticosteroids and methotrexate. Cyto-smears from the right eye AC tap revealed dispersed histiocytes and foam cells. Left eye showed non-necrotizing granuloma corresponding to the iris nodule (Figure 2A-B).

DNA sequencing of peripheral blood mononuclear cells identified a previously unreported heterozygous mutation consisting of a G>A transition in exon 4 of the *NOD2* gene (Figure 2C). The mutation resulted in a glutamic acid to lysine substitution at position 667 of the coding sequence (E667K). The E667K mutation was located in helical domain 2 of the nucleotide binding and oligomerization (NACHT) region, which is required for NOD2 funtion. The E667K is in close proximity to another mutation in the NACHT region, an asparagine to lysine substitution at position 670 (N670K) that was shown to be associated with EOS.^4^ E667 is conserved across species and therefore seems to be a critical residue (Figure 2D). It is likely that E667K inhibits, or at least reduces, the efficiency of auto-inhibition of NOD2 signaling, similar to N670K.

## DISCUSSION

*NOD2* plays a key role in innate immune response. It exists in an auto-inhibitory state due to an intramolecular interaction between the leucine-rich repeat region and the NACHT domains and undergoes activation following ligand binding.^5^ *NOD2* mutation in BS was thought to cause uncontrolled ligand-independent downstream signaling and subsequent transcription of inflammatory genes.^5^ Conversely, it has recently been demonstrated that spontaneous activation does not occur and there is decreased intracellular signaling and cytokine production in muramyl dipeptide-activated pathwayin BS.^6^

Interestingly, the first symptom in our case was intermittent febrile episodes (extra-triad manifestation) with a relatively late onset of arthritis. The presence of vitreous hemorrhage remains unexplained since no vascular abnormalities were seen. Notwithstanding, therapeutic vitrectomy helped in complete resolution of ocular inflammation even though the same could not be achieved for systemic inflammation with standard medical therapy.
